# Fibrinogen beta variants confer protection against coronary artery disease in a Greek case-control study

**DOI:** 10.1186/1471-2350-11-28

**Published:** 2010-02-18

**Authors:** Eirini V Theodoraki, Tiit Nikopensius, Julia Suhorutšenko, Vassileios Peppes, Panagiota Fili, Genovefa Kolovou, Vassileios Papamikos, Dimitrios Richter, Nikolaos Zakopoulos, Kaarel Krjutškov, Andres Metspalu, George V Dedoussis

**Affiliations:** 1 Harokopio University of Athens, Department of Dietetics and Nutritional Science, El Venizelou 70, 17671, Athens, Greece; 2 Institute of Molecular and Cell Biology, University of Tartu, Riia 23, 51010, Tartu, Estonia; 3 University of Athens Medical School "Alexandra Hospital", Department of Clinical Therapeutics, Vas. Sofias 80,11528, Athens, Greece; 4 Onassis Cardiac Surgery, 1st Cardiology Clinic, Syggrou Avenue 356, 17674, Athens, Greece; 5 Department of Cardiology, Athens Euroclinic, Athanasiadou 9, 11521, Athens, Greece; 6 Estonian Genome Center, University of Tartu, 61b Tiigi St, 50410, Tartu, Estonia; 7 Estonian Biocentre, Riia 23b, 51010, Tartu, Estonia

## Abstract

**Background:**

Although plasma fibrinogen levels are related to cardiovascular risk, data regarding the role of fibrinogen genetic variation in myocardial infarction (MI) or coronary artery disease (CAD) etiology remain inconsistent. The purpose of the present study was to investigate the effect of *fibrinogen A (FGA)*, *fibrinogen B (FGB) *and *fibrinogen G (FGG) *gene SNPs and haplotypes on susceptibility to CAD in a homogeneous Greek population.

**Methods:**

We genotyped for rs2070022, rs2070016, rs2070006 in *FGA *gene, the rs7673587, rs1800789, rs1800790, rs1800788, rs1800787, rs4681 and rs4220 in *FGB *gene and for the rs1118823, rs1800792 and rs2066865 SNPs in *FGG *gene applying an arrayed primer extension-based genotyping method (APEX-2) in a sample of CAD patients (n = 305) and controls (n = 305). Logistic regression analysis was used to calculate odds ratios (ORs) and 95% confidence intervals (CIs), before and after adjustment for potential confounders.

**Results:**

None of the *FGA *and *FGG *SNPs and *FGA, FGB, FGG *and *FGA-FGG *haplotypes was associated with disease occurrence after adjustment. Nevertheless, rs1800787 and rs1800789 SNPs in *FGB *gene seem to decrease the risk of CAD, even after adjustment for potential confounders (OR = 0.42, 95%CI: 0.19-0.90, p = 0.026 and OR = 0.44, 95%CI:0.21-0.94, p = 0.039, respectively).

**Conclusions:**

*FGA *and *FGG *SNPs as well as *FGA, FGB, FGG *and *FGA-FGG *haplotypes do not seem to be important contributors to CAD occurrence in our sample. On the contrary, *FGB *rs1800787 and rs1800789 SNPs seem to confer protection to disease onset lowering the risk by about 50% in homozygotes for the minor alleles.

## Background

Fibrinogen (Factor I) constitutes a water-soluble glycoprotein with a molecular weight of 340 kDa that is mainly synthesized in hepatocytes. It is a major factor of the coagulation system that participates in the process of hemostasis in two discrete pathways: Primarily, it is part of the final common pathway of the coagulation cascade. Secondarily, fibrinogen is bound to platelet GpIIb/IIIa membrane receptors and forms a web that provides stability to the newly-formed thrombus [1,2]. Apart from its role in coagulation reactions, fibrinogen participates in atherosclerosis development by promoting the adhesion of platelets and white blood cells to the endothelial surface [3-5] by promoting muscle cell proliferation and migration, as well as by modulating the binding of plasmin with its receptor [1]. Fibrinogen levels in plasma have been associated with coronary artery disease and myocardial infarction risk in prospective studies [6-9]. However, it is still unclear whether increased fibrinogen levels are causal to disease development or just a secondary phenomenon.

Fibrinogen circulates in plasma as a dimer, composed of three pairs of polypeptide chains denoted Aa (alpha), Bb (beta) and γ (gamma) encoded by fibrinogen alpha (FGA), beta (FGB) and gamma (FGG) genes respectively that are clustered on chromosome 4q31[10]. A variant of γ chain, named γ' is derived by alternate splicing of the primary mRNA [11]. The genes are arranged in order *FGG-FGA-FGB*, within a 50 kb region, with the transcriptional direction of *FGG *and *FGA *opposite to that of *FGB *[10].

The study of SNPs and haplotypes of fibrinogen genes in relation to coronary artery disease (CAD) and myocardial infarction (MI) occurrence has yielded to date inconsistent results. Some investigators have reported associations between fibrinogen gene SNPs or haplotypes with MI or CAD occurrence [12-14], whereas other studies have not replicated these associations [15-18].

Although numerous studies have been performed, scarce data concerning the role of fibrinogen gene SNPs or haplotypes in the Greek population are available. Therefore, we performed a retrospective case-control study involving 305 patients presenting with either CAD or acute coronary syndrome (ACS) and 305 healthy control subjects in order to investigate the impact of *FGA, FGB *and *FGG *gene SNPs and haplotypes on disease occurrence.

## Methods

Study participants were recruited from 3 hospitals found in the area of Athens. Cases were subjects presenting with either ACS or stable CAD defined as >50% stenosis in at least one of the three main coronary vessels assessed by coronary angiography. ACS was defined as acute MI or unstable angina corresponding to class III of the Braunwald classification [19]. ACS patients have also undergone coronary angiography examination that verified the presence of significant stenosis.

Controls were subjects with negative coronary angiography findings, or negative stress test, or subjects without symptoms of disease that were admitted at the same hospitals as cases and were free of any cardiovascular disease, cancer, or inflammatory diseases. Moreover, we excluded subjects with renal or hepatic disease from both study groups. The bioethics committee of Harokopio University approved the study and all participants gave their informed consent.

Regarding the clinical characteristics of study subjects, hypercholesterolemia was defined as total cholesterol levels greater than 200 mg/dl or use of hypolipidemic medication, while hypertension was defined as blood pressure levels greater than 140/90 mm Hg or use of antihypertensive medication. We classified as diabetics subjects with blood glucose levels greater than 126 mg/dl or subjects that were under special diet or treatment. Finally, positive family history of myocardial infarction was defined as the presence of myocardial infarction in first degree male relatives at age < 55 years or in first degree female relatives at age <65 years.

Altogether 13 tag SNPs were genotyped for each individual in case-control samples. Carlson's algorithm was applied for SNP selection using Tagger implementation introduced in Haploview [20], where HapMap CEU was used as a reference population (with thresholds r^2 ^≥ 0.8 and minor allele frequency (MAF) ≥ 10%) [21]. SNPs were selected for each gene including 10 kb of both upstream and downstream sequences. A SNP rs1800787 in *FGB *gene was force-included to Tagger selection referring to the previously published data. Selected SNPs, as well as their location in the gene, are presented in Table 1.

**Table 1:** TagSNPs for the *FGA, FGB *and *FGG *genes.

Gene	tagSNP	Alleles^a^	SNP location	AA change
*FGA*	rs2070022	C>T	3' UTR	-
	rs2070016	C>T	Intron 2	-
	rs2070006	A>G	5' upstream	-
*FGB*	rs7673587	C>T	5' upstream	-
	rs1800789	G>A	5' upstream	-
	rs1800790	G>A	5' upstream	-
	rs1800788	C>T	5' upstream	-
	rs1800787	C>T	5' upstream	-
	rs4681	C>T	Exon 7	Y375Y
	rs4220	G>A	Exon 8	R478K
*FGG*	rs1118823	T>A	3' downstream	-
	rs2066865	G>A	3' downstream	-
	rs1800792	T>C	5' upstream	-

^a^Alleles are depicted from the coding strand for all SNPs except of rs2070022, rs2070016, rs2070006, rs2066865 and rs1800792 that are depicted from the non-coding one.

Genomic DNA was extracted from whole blood using the salting-out method [22]. Genotyping was performed using an arrayed primer extension-based genotyping method (APEX-2). This method allows multiplex DNA amplification and detection of SNPs on microarrays via four-color single-base primer extension [23].

The standard chi-square test was used to test for deviations from Hardy-Weinberg equilibrium and to evaluate the differences in genotype distributions between cases and controls.

The odds ratios (ORs) were calculated using logistic regression under the assumption of the additive, dominant and recessive models. Logistic regression including age, sex, and the presence of diabetes, hypertension, hypercholesterolemia and smoking as covariates was used to calculate adjusted ORs. Analysis of single SNPs effects was performed using PLINK 1.05 [24].

**Figure 1:**
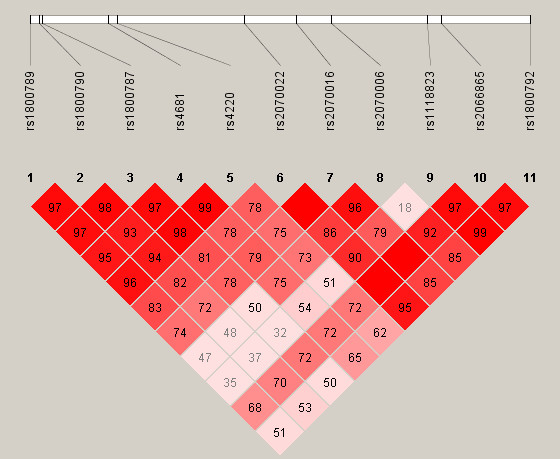
**Linkage disequilibrium structure**. D' values between SNPs in FGA, FGB and FGG genes.

Haplotypes were constructed for each gene separately and for *FGG-FGA *genes. We did not consider *FGA-FGG-FGB *haplotypes in the analysis due to weak LD (Linkage Disequilibrium) between *FGA-FGG *and *FGB *variants. THESIAS software [25] was used to calculate haplotype frequencies in cases and controls as well as ORs and respective 95% confidence intervals before and after adjustment for the aforementioned covariates using the most common haplotype as a reference. LD measures (r^2 ^and D') between SNPs were calculated using Haploview [20]. D' values are given in Figure 1. All p-values are based on two-sided tests and compared to a significance level of 5%.

Power analysis was performed using Quanto 1.2 software.

## Results

Table 2 summarises the characteristics of 305 CAD and ACS cases and 305 control subjects. The two study groups differed in a predictable manner, i.e. cases exhibited a higher prevalence of risk factors such as hypercholesterolemia, hypertension, diabetes and smoking. Patients presenting ACS represented 62.6% of all cases. Mean age, as well as the percentage of male subjects was lower among controls than in cases. BMI did not present statistically significant differences between the two study groups. Although hypercholesterolemia was most common among cases, total cholesterol and LDL levels are higher in the control group, due to the less frequent use of hypolipidemic medication.

**Table 2:** General characteristics of patients and controls included in our study.

Subject characteristics	Cases (n = 305)	Controls (n = 305)	*P*-value
**Stable CAD (%)**	37.4	-	
**ACS (%)**	62.6	-	
**Age (years)**	63.14 ± 11.41	60.37 ± 14.86	0.011
**Male sex (%)**	81.6	70.2	0.001
**BMI (kg/m^2^)**	27.9 ± 3.8	28.2 ± 4.6	0.403
**Hypercholesterolemia (%)**	76.9	59.0	<0.001
**Diabetes (%)**	32.4	15.1	<0.001
**Hypertension (%)**	71.7	58.8	0.001
**Family history of MI (%)**	28.0	16.7	0.001
**Current or former smoking (%)**	74.2	61.0	<0.001
**Total cholesterol (mg/dl)**	197.7 ± 47.7	214.4 ± 40.4	<0.001
**LDL cholesterol (mg/dl)**	126.6 ± 41.6	141.2 ± 36.4	<0.001
**HDL cholesterol (mg/dl)**	50.6 ± 12.6	45.8 ± 14.3	<0.001
**Triglycerides (mg/dl)**	144.2 ± 66.8	118.1 ± 65.6	<0.001

All SNPs were in Hardy-Weinberg equilibrium except for FGB rs7673587 and rs1800788 SNPs (p < 0.05) that were excluded from subsequent analysis.

Logistic regression analysis was performed for *FGA, FGB *and *FGG *gene SNPs separately, under the assumption of the additive, dominant and recessive models. ORs and 95% CIs were calculated before and after adjustment for age, sex and the presence of hypercholesterolemia, hypertension, diabetes and smoking. Four SNPs were nominally associated with disease in at least one model of inheritance (Table 3).

**Table 3:** Results from logistic regression analysis for SNPs with significant associations before adjustment.

GENE	SNP	Genotype frequency			Minor allele frequency		Model	OR;95% CI	*P*-value	**adjusted OR; 95% CI**^a^	**adjusted *P*-value**^a^
			Cases	Controls	Cases	Controls					
		AA	0.36	0.46			Additive	1.26;1.00-1.59	**0.055**	**1.26;0.97-1.63**	**0.081**
*FGA*	rs2070006	AG	0.51	0.41	0.39	0.34	Dominant	1.51;1.09-2.09	**0.013**	**1.39;0.97-1.99**	**0.077**
		GG	0.13	0.13			Recessive	1.07;0.67-1.70	0.788	1.28;0.77-2.14	0.336
		GG	0.56	0.58			Additive	0.92; 0.71-1.19	0.514	0.88;0.66-1.17	0.376
*FGB*	rs1800789	GA	0.39	0.33	0.24	0.26	Dominant	1.06; 0.77-1.46	0.733	1.00;0.70-1.44	0.990
		AA	0.05	0.09			Recessive	0.47; 0.24-0.92	**0.026**	0.42;0.19-0.90	**0.026**
		CC	0.58	0.55			Additive	0.83; 0.64-1.07	0.153	0.77;0.58-1.03	0.076
*FGB*	rs1800787	CT	0.37	0.36	0.24	0.27	Dominant	0.89; 0.65-1.23	0.473	0.81;0.57-1.16	0.254
		TT	0.05	0.09			Recessive	0.50; 0.26-0.96	**0.039**	0.44;0.21-0.94	**0.034**
		GG	0.61	0.70			Additive	1.31;0.99-1.75	0.062	1.32;0.97-1.80	0.077
*FGG*	rs2066865	GA	0.34	0.26	0.21	0.17	Dominant	1.40;1.00-1.97	**0.051**	1.42;0.98-2.07	**0.066**
		AA	0.05	0.04			Recessive	1.31;0.59-2.93	0.513	1.35;0.58-3.12	0.486

Results from logistic regression analysis for *FGA, FGB *and *FGG *gene SNPs that were significantly associated with disease, before adjustment for confounding variables.

^a ^adjustment for age, sex and the presence of hypercholesterolemia, hypertension, diabetes and smoking

Thus, carriers of one minor allele of rs2070006 in *FGA *gene exhibited an OR of 1.26 (95% CI:0.99-1.59, p = 0.055), that after adjustment was 1.26 (95% CI:0.98-1.63, p = 0.081). When carriers of the minor allele were grouped together, i.e. when modeled dominantly, the unadjusted OR was 1.51 (95% CI: 1.09-2.09, p = 0.013), while after adjustment OR was 1.39 (95% CI:0.97-1.99, p = 0.077).

In the case of *FGB *gene two SNPs, rs1800787 and rs1800789, were associated with disease occurrence in the recessive model, and the association remained significant after adjustment for the confounding variables (OR = 0.47, 95% CI:0.24-0.92, p = 0.026 and OR = 0.50, 95% CI:0.27-0.96, p = 0.039, respectively). The association of those SNPs with disease was significant even after further adjustment for obesity (BMI > 27) (OR = 0.40, 95% CI:0.18-0.18, p = 0.023 and OR = 0.38, 95% CI:0.17-0.85, p = 0.019, respectively).

Rs2066865 in *FGG *gene showed borderline association with CAD. For carriers of one minor allele, the unadjusted OR was 1.31 (95% CI:0.99-1.75, p = 0.062), while after adjustment it was 1.32 (95% CI:0.97-1.80, p = 0.077). Results did not differ much when the dominant model was considered. No other SNP in FGA, FGB and FGG genes was associated with disease occurrence.

In Table 4 the inferred haplotypes for *FGA, FGB, FGG *and *FGA-FGG *SNPs with frequency >5% are presented. In Table 5 the ORs and 95% CIs are presented for each haplotype before and after adjustment for the same confounding variables as in the case of single SNPs. We used the most common haplotype as a reference category. *FGA-FGG *H3 haplotype TGATTA bearing the 2 minor alleles of rs2070006 and rs2066865 was associated with CAD in the unadjusted analysis (OR = 1.42, 95% CI:1.02-1.98, p = 0.040), but after adjustment for confounding factors the statistical significance was lost (OR = 1.38, 95% CI: 0.94-2.02, p = 0.098).

**Table 4:** Haplotypes for *FGA, FGB, FGG *and *FGA-FGG *gene SNPs with frequencies >5%.

SNPs											
Haplotypes	rs2070006	rs2070022	rs2070016	rs1800792	rs1118823	rs2066865	rs1800787	rs1800789	rs1800790	rs4681	rs4220
FGA-H1	**T**	G	A								
FGA-H2	C	G	A								
FGA-H3	C	G	**G**								
FGA-H4	C	**A**	A								
FGG-H1				**C**	T	G					
FGG-H2				T	**A**	G					
FGG-H3				T	T	**A**					
FGB-H1							**C**	**G**	**G**	C	G
FGB-H2							T	A	A	**T**	**A**
FGA-FGG-H1	C	G	A	**C**	T	G					
FGA-FGG-H2	C	G	**G**	**C**	T	G					
FGA-FGG-H3	**T**	G	A	T	T	**A**					
FGA-FGG-H4	C	**A**	A	T	**A**	G					
FGA-FGG-H5	T	**A**	**G**	T	**A**	G					

Bold and underlined letters indicate SNP minor alleles.

**Table 5:** Frequencies, ORs and 95% CIs for CAD in relation to the most frequent haplotypes.

Haplotype	Controls (%)	Cases (%)	OR;95%CI	*P*-value	**adjusted OR;95%CI**^a^	**adjusted *P*-value**^a^
FGA-H1	33.00	38.13	*referent*		*referent*	
FGA-H2	28.88	26.25	0.80;0.60-1.05	0.104	0.81;0.59-1.11	0.186
FGA-H3	21.10	20.27	0.84;0.62-1.14	0.256	0.75;0.53-1.07	0.111
FGA-H4	16.20	14.65	0.78;0.55-1.11	0.164	0.84;0.56-1.26	0.396
FGG-H1	48.84	45.16	*referent*		*referent*	
FGG-H2	32.78	32.81	1.06;0.83-1.36	0.638	1.13;0.86-1.50	0.387
FGG-H3	16.55	20.78	1.31;0.97-1.77	0.078	1.31;0.93-1.87	0.122
FGB-H1	72.34	75.37	*referent*		*referent*	
FGB-H2	21.84	20.77	0.92;0.71-1.20	0.547	0.89;0.65-1.21	0.463
FGA-FGG-H1	26.00	23.97	*referent*		*referent*	
FGA-FGG-H2	19.50	18.31	1.05;0.76-1.45	0.771	0.94;0.65-1.37	0.755
FGA-FGG-H3	15.71	20.08	1.42;1.02-1.98	**0.040**	1.38;0.94-2.02	0.098
FGA-FGG-H4	15.85	14.60	1.03;0.71-1.48	0.893	1.09;0.72-1.66	0.665
FGA-FGG-H5	14.03	15.28	1.22;0.86-1.75	0.264	1.21;0.79-1.84	0.381

^a ^adjustment for age, sex and the presence of hypercholesterolemia, hypertension, diabetes and smoking

Further adjustment for lipid levels, i.e. total cholesterol, LDL cholesterol and HDL cholesterol, did not significantly affect our results concerning both single SNP and haplotype effects.

## Discussion

Evidence from epidemiologic studies and meta-analyses suggests that increased plasma fibrinogen levels are related to increased coronary artery disease risk [6]. Despite the existence of numerous studies that support an association between plasma fibrinogen levels and certain SNPs or haplotypes [13,15,26-28], data linking the latter with CAD occurrence still remain controversial.

In the present study we used a tagSNP approach to evaluate the role of genetic variation across *FGA, FGB *and *FGG *genes in the occurrence of coronary artery disease in a homogeneous Greek population sample of 305 patients and 305 controls. CAD was defined as either presence of angiographically proven significant stenosis in coronary vessels or ACS.

The allele frequencies in controls, observed in our study, were similar to the ones reported for HapMap CEU reference population. Rs2070006 and rs2066865 SNPs in *FGA *and *FGG *genes respectively were nominally associated with increased risk of CAD both in the additive and dominant models of inheritance, but statistical significance was lost after adjustment. On the other hand, homozygotes for the minor alleles of rs1800787 and rs1800789 SNPs in *FGB *gene exhibited a decreased risk of CAD remaining statistically significant after adjustment for confounding factors.

Haplotype analysis showed that when *FGA *and *FGG *gene SNPs were considered together, *FGA-FGG*-H3 haplotype TGATTA bearing the minor alleles of both rs2070006 and rs2066865 SNPs was associated with an increase in disease risk in the unadjusted analysis, but after adjustment this association disappeared.

Therefore, our results do not reveal an important role of *FGA *and *FGG *gene SNPs and *FGA, FGB, FGG *and *FGA-FGG *haplotypes in CAD occurrence and are in accordance with previously published data. The initial associations published by Mannila et al. [12-14] supporting a role of *FGG-FGA *and *FGG-FGB *haplotypes in myocardial infarction risk have not been replicated by other investigators that studied single SNPs and haplotypes in *FGA, FGB *and *FGG *genes in MI or CAD phenotypes [15-18].

Rs1800790 SNP in *FGB *gene was shown to be associated with decreased MI risk applying the recessive model in a meta-analysis [29]. Recently, the same SNP was found to exert protective effect against premature myocardial infarction in a Greek population [30]. In our study we could not replicate these findings. Interestingly, we found rs1800787 and rs1800789 variants, that are highly correlated with rs1800790 (r^2 ^= 0.943 and r^2 ^= 0.928, respectively), to decrease disease risk by about 50%, when modeled recessively. One could hypothesize that the effect of rs1800790, found in the previous studies, is attributed to the strong LD with SNPs rs1800787 and/or rs1800789 - these two tightly linked SNPs most likely are representing the same signal of association with the disease and either of these SNPs might be the functional one. Previous studies that included these SNPs, or others that are in strong LD with them, resulted in negative findings, but in those studies the effect of the SNPs in the recessive model was not considered [15,16].

Our study is limited by the small sample size. A posterior analysis revealed that the power of our sample to detect an odds ratio from 0.4-0.5 was 0.5-0.8, depending on minor allele frequency, with significance level alpha 0.05. We cannot exclude the possibility of a modest effect of SNPs or haplotypes in disease predisposition that would possibly be apparent in a larger sample. Nevertheless, our sample size is similar to that of Mannila et al. that studied eight fibrinogen SNPs in 377 post-infarction patients and 387 healthy individuals and found that fibrinogen haplotypes, and not SNPs, were associated with the risk of MI [14].

Another important limitation of our study is the lack of information concerning the effect of SNPs or haplotypes on fibrinogen levels. Moreover, we have not taken into account the effect of proinflammatory markers, such as IL-6, that have been shown to modify the effect of SNPs on fibrinogen levels [26], and possibly the effect of SNPs in disease risk. Nevertheless, in the multivariate analysis we have adjusted our models for the presence of obesity, hypertension, hypercholesterolemia and diabetes that represent in a way the proinflammatory status and may partially compensate for the lack of information for the levels of specific inflammatory markers.

The results of our study should be interpreted with caution, taking into account the multiple tests performed. If we applied the conservative Bonferroni's correction then the level of statistical significance should be 0.001 and none of our associations would remain significant. Nevertheless, the fact that rs1800787 and rs1800789 are highly correlated with rs1800790 that has been previously associated with disease increases our confidence for our results.

Futhermore, we cannot exclude the possibility of misclassification of subjects with silent CAD in the control group among subjects that were not subjected to coronary angiography or stress test and who reported absence of symptoms of disease. Finally, cases with fatal MI were not included. Thus, we cannot rule out the possibility that this polymorphism may predispose for more severe disease phenotypes.

## Conclusions

The results of the present study suggest that *FGA *and *FGG *variants as well as *FGA*, *FGB, FGG *and *FGA-FGG *haplotypes do not seem to be important contributors to CAD occurence in our Greek population. Nevertheless, *FGB *rs1800787 and rs1800789 variants, in the recessive model, seem to confer protection to disease onset lowering the risk by about 50%.

## Competing interests

The authors declare that they have no competing interests.

## Authors' contributions

EVT participated in study design, sample recruitment, DNA isolation, statistical analysis, interpretation of data and drafted the manuscript. TN participated in SNP selection, genotyping and manuscript revision. JS participated in genotyping. VP participated in sample recruitment and helped to draft the manuscript. PF participated in sample recruitment and DNA isolation. GK participated in sample recruitment and manuscript revision. VP participated in sample recruitment and DNA isolation. DR and NZ participated in sample recruitment. AM coordinated SNP selection and genotyping. GVD was the general coordinator of the study.

All authors read and approved the final manuscript.

## Pre-publication history

The pre-publication history for this paper can be accessed here:

http://www.biomedcentral.com/1471-2350/11/28/prepub

## References

[B1] Koenig W Fibrin(ogen) in cardiovascular disease: an update Thromb Haemost 2003 89 4 601 609 12669113

[B2] Reinhart WH Fibrinogen--marker or mediator of vascular disease? Vasc Med 2003 8 3 211 216 10.1191/1358863x03vm494ra 14989564

[B3] Duperray A Languino LR Plescia J McDowall A Hogg N Craig AG Berendt AR Altieri DC Molecular identification of a novel fibrinogen binding site on the first domain of ICAM-1 regulating leukocyte-endothelium bridging J Biol Chem 1997 272 1 435 441 10.1074/jbc.272.1.435 8995280

[B4] Harley SL Sturge J Powell JT Regulation by fibrinogen and its products of intercellular adhesion molecule-1 expression in human saphenous vein endothelial cells Arterioscler Thromb Vasc Biol 2000 20 3 652 658 10712387 10.1161/01.atv.20.3.652

[B5] Stolpe A van de Jacobs N Hage WJ Tertoolen L van Kooyk Y Novakova IR de Witte T Fibrinogen binding to ICAM-1 on EA.hy 926 endothelial cells is dependent on an intact cytoskeleton Thromb Haemost 1996 75 1 182 189 8713799

[B6] Danesh J Collins R Appleby P Peto R Association of fibrinogen, C-reactive protein, albumin, or leukocyte count with coronary heart disease: meta-analyses of prospective studies JAMA 1998 279 18 1477 1482 10.1001/jama.279.18.1477 9600484

[B7] Danesh J Lewington S Thompson SG Lowe GD Collins R Kostis JB Wilson AC Folsom AR Wu K Benderly M Plasma fibrinogen level and the risk of major cardiovascular diseases and nonvascular mortality: an individual participant meta-analysis JAMA 2005 294 14 1799 1809 10.1001/jama.294.14.1799 16219884

[B8] Ernst E Resch KL Fibrinogen as a cardiovascular risk factor: a meta-analysis and review of the literature Ann Intern Med 1993 118 12 956 963 8489110 10.7326/0003-4819-118-12-199306150-00008

[B9] Maresca G Di Blasio A Marchioli R Di Minno G Measuring plasma fibrinogen to predict stroke and myocardial infarction: an update Arterioscler Thromb Vasc Biol 1999 19 6 1368 1377 10364066 10.1161/01.atv.19.6.1368

[B10] Kant JA Fornace AJ Jr Saxe D Simon MI McBride OW Crabtree GR Evolution and organization of the fibrinogen locus on chromosome 4: gene duplication accompanied by transposition and inversion Proc Natl Acad Sci USA 1985 82 8 2344 2348 10.1073/pnas.82.8.2344 2986113 PMC397554

[B11] Chung DW Davie EW gamma and gamma' chains of human fibrinogen are produced by alternative mRNA processing Biochemistry 1984 23 18 4232 4236 10.1021/bi00313a033 6091742

[B12] Mannila MN Eriksson P Ericsson CG Hamsten A Silveira A Epistatic and pleiotropic effects of polymorphisms in the fibrinogen and coagulation factor XIII genes on plasma fibrinogen concentration, fibrin gel structure and risk of myocardial infarction Thromb Haemost 2006 95 3 420 427 16525568 10.1160/TH05-11-0777

[B13] Mannila MN Eriksson P Leander K Wiman B de Faire U Hamsten A Silveira A The association between fibrinogen haplotypes and myocardial infarction in men is partly mediated through pleiotropic effects on the serum IL-6 concentration J Intern Med 2007 261 2 138 147 10.1111/j.1365-2796.2006.01749.x 17241179

[B14] Mannila MN Eriksson P Lundman P Samnegard A Boquist S Ericsson CG Tornvall P Hamsten A Silveira A Contribution of haplotypes across the fibrinogen gene cluster to variation in risk of myocardial infarction Thromb Haemost 2005 93 3 570 577 15735812 10.1160/TH04-10-0698

[B15] Carty CL Cushman M Jones D Lange LA Hindorff LA Rice K Jenny NS Durda JP Walston J Carlson CS Associations between common fibrinogen gene polymorphisms and cardiovascular disease in older adults. The Cardiovascular Health Study Thromb Haemost 2008 99 2 388 395 18278190 10.1160/TH07-08-0523

[B16] Kardys I Uitterlinden AG Hofman A Witteman JC de Maat MP Fibrinogen gene haplotypes in relation to risk of coronary events and coronary and extracoronary atherosclerosis: the Rotterdam Study Thromb Haemost 2007 97 2 288 295 17264959

[B17] Koch W Hoppmann P Biele J Mueller JC Schomig A Kastrati A Fibrinogen genes and myocardial infarction: a haplotype analysis Arterioscler Thromb Vasc Biol 2008 28 4 758 763 10.1161/ATVBAHA.107.157842 18202324

[B18] Uitte de Willige S de Visser MC Houwing-Duistermaat JJ Rosendaal FR Vos HL Bertina RM Genetic variation in the fibrinogen gamma gene increases the risk for deep venous thrombosis by reducing plasma fibrinogen gamma' levels Blood 2005 106 13 4176 4183 10.1182/blood-2005-05-2180 16144795

[B19] Braunwald E Shattuck lecture--cardiovascular medicine at the turn of the millennium: triumphs, concerns, and opportunities N Engl J Med 1997 337 19 1360 1369 10.1056/NEJM199711063371906 9358131

[B20] Barrett JC Fry B Maller J Daly MJ Haploview: analysis and visualization of LD and haplotype maps Bioinformatics 2005 21 2 263 265 10.1093/bioinformatics/bth457 15297300

[B21] Carlson CS Eberle MA Rieder MJ Yi Q Kruglyak L Nickerson DA Selecting a maximally informative set of single-nucleotide polymorphisms for association analyses using linkage disequilibrium Am J Hum Genet 2004 74 1 106 120 10.1086/381000 14681826 PMC1181897

[B22] Miller SA Dykes DD Polesky HF A simple salting out procedure for extracting DNA from human nucleated cells Nucleic Acids Res 1988 16 3 1215 10.1093/nar/16.3.1215 3344216 PMC334765

[B23] Krjutskov K Andreson R Magi R Nikopensius T Khrunin A Mihailov E Tammekivi V Sork H Remm M Metspalu A Development of a single tube 640-plex genotyping method for detection of nucleic acid variations on microarrays Nucleic Acids Res 2008 36 12 e75 10.1093/nar/gkn357 18539607 PMC2475630

[B24] Purcell S Neale B Todd-Brown K Thomas L Ferreira MA Bender D Maller J Sklar P de Bakker PI Daly MJ PLINK: a tool set for whole-genome association and population-based linkage analyses Am J Hum Genet 2007 81 3 559 575 10.1086/519795 17701901 PMC1950838

[B25] Tregouet DA Barbaux S Escolano S Tahri N Golmard JL Tiret L Cambien F Specific haplotypes of the P-selectin gene are associated with myocardial infarction Hum Mol Genet 2002 11 17 2015 2023 10.1093/hmg/11.17.2015 12165563

[B26] Jacquemin B Antoniades C Nyberg F Plana E Muller M Greven S Salomaa V Sunyer J Bellander T Chalamandaris AG Common genetic polymorphisms and haplotypes of fibrinogen alpha, beta, and gamma chains affect fibrinogen levels and the response to proinflammatory stimulation in myocardial infarction survivors: the AIRGENE study J Am Coll Cardiol 2008 52 11 941 952 10.1016/j.jacc.2008.06.016 18772067

[B27] Tybjaerg-Hansen A Agerholm-Larsen B Humphries SE Abildgaard S Schnohr P Nordestgaard BG A common mutation (G-455--> A) in the beta-fibrinogen promoter is an independent predictor of plasma fibrinogen, but not of ischemic heart disease. A study of 9,127 individuals based on the Copenhagen City Heart Study J Clin Invest 1997 99 12 3034 3039 10.1172/JCI119499 9185528 PMC508156

[B28] van 't Hooft FM von Bahr SJ Silveira A Iliadou A Eriksson P Hamsten A Two common, functional polymorphisms in the promoter region of the beta-fibrinogen gene contribute to regulation of plasma fibrinogen concentration Arterioscler Thromb Vasc Biol 1999 19 12 3063 3070 10591688 10.1161/01.atv.19.12.3063

[B29] Boekholdt SM Bijsterveld NR Moons AH Levi M Buller HR Peters RJ Genetic variation in coagulation and fibrinolytic proteins and their relation with acute myocardial infarction: a systematic review Circulation 2001 104 25 3063 3068 10.1161/hc5001.100793 11748101

[B30] Rallidis LS Gialeraki A Fountoulaki K Politou M Sourides V Travlou A Lekakis I Kremastinos DT G-455A polymorphism of beta-fibrinogen gene and the risk of premature myocardial infarction in Greece Thromb Res 2009 19409601 10.1016/j.thromres.2009.02.017

